# Small bowel schwannoma revealed during an inguinal hernia: a case report

**DOI:** 10.1186/1752-1947-8-287

**Published:** 2014-08-26

**Authors:** Aziz Zentar, Youssef Tijani, Hakim Elkaoui, Jihad Elghanmi, Khalid Sair, Mustafa Taberkant, Hassan Taoufik Chtata

**Affiliations:** 1 Department of Digestive Surgery, Mohammed V Military Hospital, Mohammed V University of Rabat, Rabat, Morocco; 2 Department of Vascular Surgery, Mohammed V Military Hospital, Mohammed V University of Rabat, Street corner souss-qahira, Nuild 16, N°6 Kenitra, Rabat, Morocco; 3 Department of Urology B, Avicenne University Hospital, Rabat, Morocco

**Keywords:** Inguinal hernia, Schwannoma, Small bowel

## Abstract

**Introduction:**

The association of bowel tumor and inguinal hernia is rare. We report according to our research the first case of the migration of a small bowel schwannoma into an inguinal hernia.

**Case presentation:**

We report the case of a 51-year-old Moroccan malen admitted for a non-reducible right inguinal hernia in which surgical exploration showed the presence of a small bowel tumor that had migrated into his hernia sac. A histopathological examination of the tumor was in favor of a small bowel schwannoma.

**Conclusion:**

Small bowel schwannoma is an exceptional clinical entity for which the diagnosis is difficult; its confirmation needs histological and immunohistochemical studies.

## Introduction

The migration of an intestinal tumor into inguinal hernia is a very rare entity. However the migration of a schwannoma of the small bowel into inguinal hernia is exceptional.

We report according to our research the first case of migration of small bowel schwannoma into an inguinal hernia.

## Case presentation

We report the case of a 51-year-old Moroccan man with no past medical history who presented with a right inguinal mass looking like an inguinal hernia.

The clinical history started 1 year ago with the appearance of a right inguinal mass, impulsive during cough and reducible.

He observed an increase in the mass volume, and the appearance of a second hard and non-reducible mass 3 months later.

**Figure 1:**
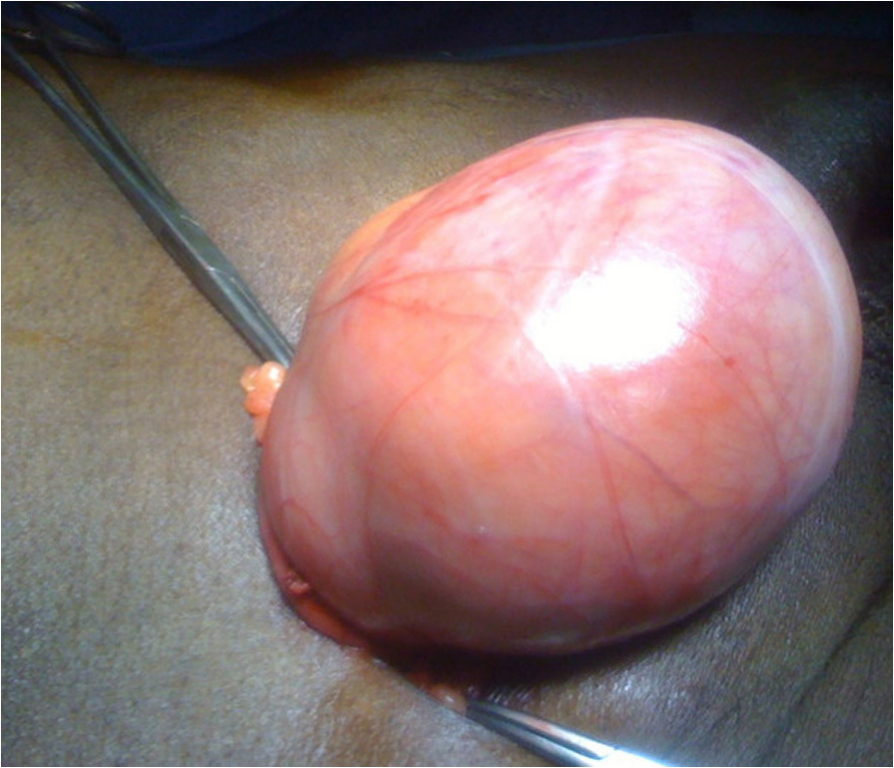
Operative view showing a 10cm mass located on the bowel.

A clinical examination showed the presence of two inguinal masses the former soft and reducible and the latter hard and non-reducible. There was no associated nausea or vomiting. He had a history of hypertension for the previous 9 years. He had no significant changes in standard biochemical findings. The first diagnosis was the migration of his bowel in the hernia sac.During surgical exploration we found an indirect inguinal hernia; however, at the opening of the sac we discovered a 10cm mass originating from the ileal portion of his bowel tract (Figure 1).

**Figure 2:**
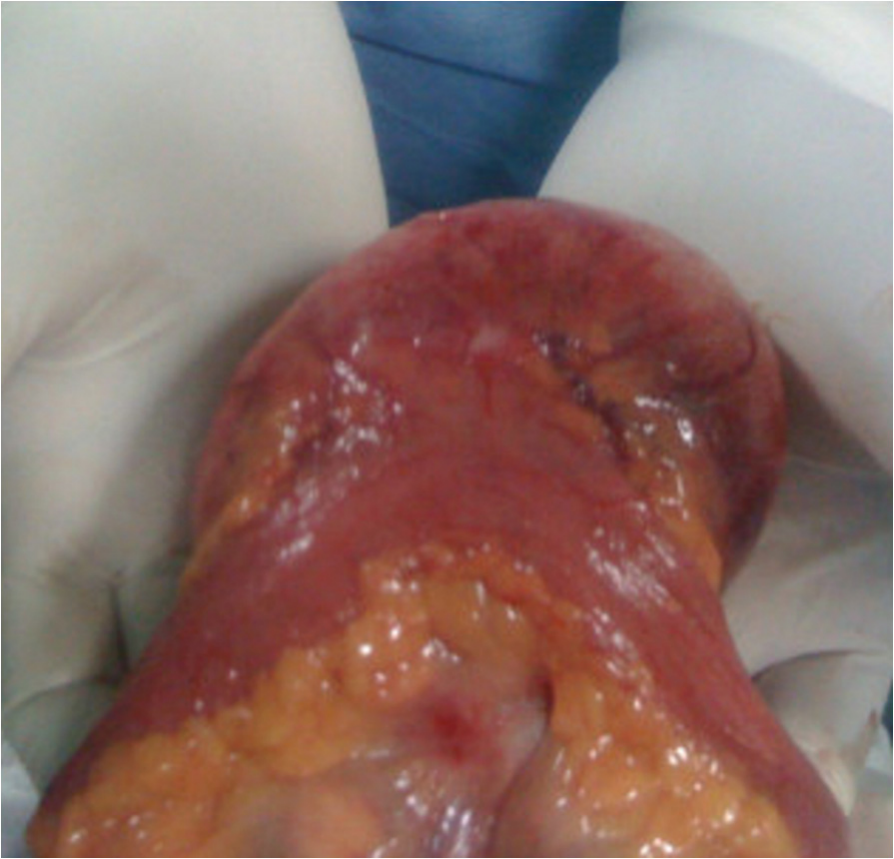
Operative view showing the mass is attached to the antimesenteric edge.

**Figure 3:**
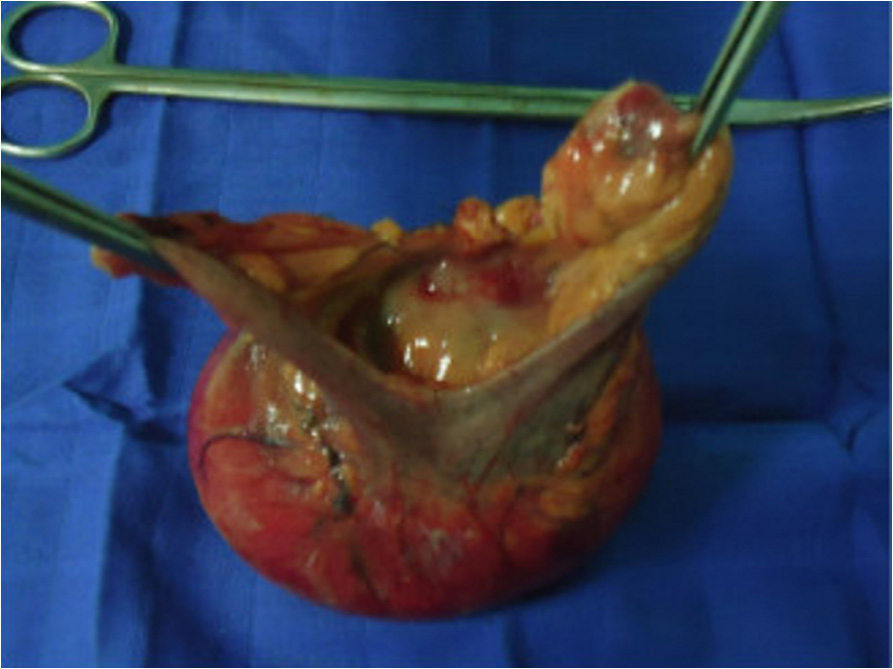
View showing the mass after resection.

We could not find any other mass in the examination of the remaining parts of his digestive system.Our surgery consisted of the resection of a reddish well-encapsulated tumor, measuring 10cm in diameter, originating from the antimesenteric border of the small bowel, without obstruction of its lumen leaving a 6cm (Figures 2 and 3) safety margin from both sides of the termino-terminal anastomosis.

**Figure 4:**
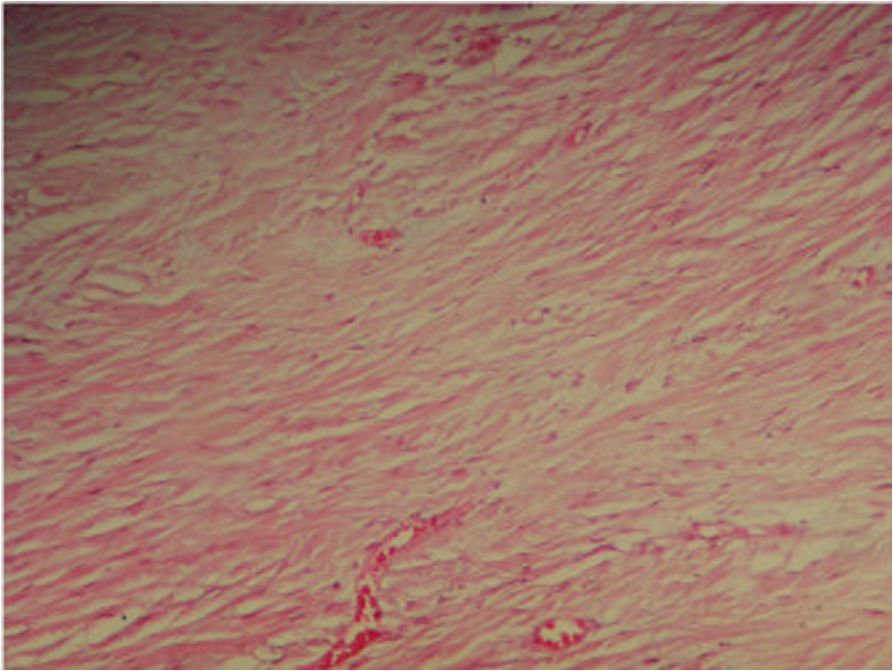
Anatomopathologic aspect of bowel schwannoma.

Hernia treatment was done according to the Lichtenstein procedure.An anatomopathological study showed the presence of fusiform cells, in which the immunochemical study demonstrated cells expression of antibodies peroxidase and the immunostaining of cells was in favor of anti-PS100, and the non-expression of anti-cluster of differentiation (CD) 117 and anti-desmin, hence proving its neurological nature schwannoma (Figure 4).

## Discussion

The content of 10% of inguinal hernias is the bowel system [1]. The literature reveals that hernias may also contain different abdominal organs, such as urological and gynecological organs [2,3]; however less than 0.5% of hernias contain a tumor [4]. According to Lejar’s classification of primary tumors in the hernia sac, we may differentiate two types: saccular and intrasaccular [5]. Saccular tumors involve the hernia sac such as mesothelioma; intrasaccular tumors involve organs inside the sac. The majority of intrasaccular tumors are of intestinal origin.

In a series of 22,000 inguinal hernioplasties described by Nicholson *et al.*[6], they found that the incidence of tumor metastasis into the inguinal region is 0.07% of which 40% of the tumor metastases are secondary to intestinal tumor. The coexistence of an intra-abdominal mass and inguinal hernia was also reported [6-10], one possible explanation for this is the increased intra-abdominal pressure secondary to the presence of an intra-abdominal tumor, particularly in older patients [9,11]. In our case report, the histopathological study was in favor of bowel schwannoma. Schwannoma localization in the bowel tract is rare and not frequently described in the literature, and it belongs to tumors of the bowel system which include digestive stromal tumors and differentiated tumors, which are less frequent [12]; the preferential localization of digestive tube schwannoma is the stomach [13,14]. Other localizations are described but are rare: colon, rectum and esophagus [15-17]. The clinical presentation of this tumor is not specific, frequently the patient complains of dyspepsia and epigastric pain [13,14,18]. In lower localization, the patient may complain of severe constipation and rectorrhagia [15]. The age of the patient varies from 18- to 87-years old, with a mean age of 65 years [15]; a slight predominance in women is found. Tumor [13-16] size varies from 0.5 to 11cm [18] and giant schwannoma remain exceptional [17]. Malignant schwannoma localization in the digestive tract is not described [19].

In general, at endoscopic examination, the tumor appears submucosal with a large protrusion into the lumen of the digestive tube associated to small erosions of the mucosa. A biopsy is usually negative [20] and the diagnosis is based on the anatomopathological study of the removed tumor. In immunostaining studies schwannomas are always positive for proteins S100, positive for glial fibrillary acidic protein and CD57 marker but less constant and always negative for muscle markers [14]. The treatment of colorectal schwannoma is segmental colectomy, hemicolectomy or enucleation [15]. In fact, we could not find the association between Von Recklinghausen disease and the patient having digestive schwannoma [13-15,21].

We should differentiate between schwannomas and digestive stromal tumors. The latter are rare; they frequently originate from the connective system of the digestive tube [21].

Four types are identified [13]:

– Smooth muscle cells tumor.

– Nervous system tumor.

– Nervous and smooth muscle cells tumors.

– Undifferentiated tumor.

Stomach and small bowel are the most frequent localization.

All submucosal tumors of the digestive layer are considered stromal tumors; neither endoscopy nor echoendoscopy may confirm the diagnosis, only histological examination and immunohistochemical study may give us the precise diagnosis. Digestive stromal tumors show structural and immunophenotypic heterogeneity according to the segment in the digestive tube, and in the same tumor, the expression of CD117 is frequent but less constant [11].

## Conclusions

The association of inguinal hernia and bowel schwannoma is exceptional; the prognosis is favorable.

The main difficulty is in precise diagnosis and for this histological and immunohistochemical studies are necessary.

## Consent

Written informed consent was obtained from the patient for publication of this case report and any accompanying images. A copy of the written consent is available for review by the Editor-in-Chief of this journal.

## Abbreviations

ANTI PS 100: Antibodies peroxidase immunostaining 100; CD: Cluster of differentiation.

## Competing interests

The authors declare that they have no competing interests.

## Authors’ contributions

AZ reviewed our patient’s case, data and figures, and was a major contributor in writing the manuscript. HC and YT reviewed our patient’s case and data, completed subsequent drafts of the manuscript and were major contributors in writing the manuscript. HE and JE were involved during the initial presentation of our patient’s case and were major contributors in writing the manuscript. KS was involved during the initial presentation of our patient’s case. MT reviewed our patient’s case, data, coordinated the authors and was a major contributor in writing the manuscript. All authors read and approved the final manuscript.

## References

[B1] McFayden BV Mathis CR Inguinal herniorrhaphy: complications and recurrences Semin Laparosc Surg 1994 1 128 140 10401048 10.1053/SLAS00100128

[B2] Habib FA McAleese P Kolachalam RB Laparoscopic approach to the management of incarcerated hernia of appendices epiploicae: report of two cases and review of the literature Surg Laparosc Endosc 1998 8 425 428 10.1097/00019509-199812000-00005 9864108

[B3] Oruc MT Akbulut Z Ozozan O Coskun F Urological findings in inguinal hernias: a case report and review of the literature Hernia 2004 8 76 79 10.1007/s10029-003-0157-6 13680305

[B4] Yoell JH Surprises in inguinal hernial sacs, diagnosis of tumors by microscopic examination Calif Med 1959 91 146 148 13846556 PMC1577810

[B5] Lejar J Neoplasmesherniairesetperi-herniaires Gaz Hosp 1889 62 801

[B6] Nicholson CP Donohue JH Thompson GB Lewis JE A study of metastatic cancer found during inguinal hernia repair Cancer 1992 69 3008 3011 10.1002/1097-0142(19920615)69:12<3008::AID-CNCR2820691224>3.0.CO;2-8 1591694

[B7] Hale MDA Solla MJA Complete colonic obstruction caused by a sigmoid colon cancer incarcerated in an inguinal hernia sac South Med J 1991 84 1280 1281 10.1097/00007611-199110000-00032 1925737

[B8] Lowenfels AB Ahmed N Rohman M Lefkowitz M Hernia-sac cancer Lancet 1969 I 651 10.1016/s0140-6736(69)92013-3 4179886

[B9] Terezis NL Dawis WC Jackson FC Carcinoma of colon associated with inguinal hernia New Eng J Med 1963 268 774 776 10.1056/NEJM196304042681407 13980652

[B10] Wlodarczyk A Bielecki K Ciesielski A Kozicki I Coexistence of left inguinal hernia and left colon cancer – a case report and literature review Mater Med Pol 1996 28 33 34 9088124

[B11] Geelhoed GW Millar RC Ketcham AS Hernia presentation of cancer in the groin Surgery 1974 75 436 441 4811340

[B12] Bedossa P Martin E Quoi de neufsur les tumeursconjonctives du tube digestif? Ann Pathol 1994 14 350 356 7818737

[B13] Prevot S Bienvenu L Vaillant JC Saint-Maur PP Benign schwannoma of the digestive tract: a clinicopathologic and immunohistochemical study of five cases, including a case of esophageal tumor Am J Surg Pathol 1999 23 431 436 10.1097/00000478-199904000-00007 10199472

[B14] Sarlomo-Rikala M Miettinen M Gastric schwannoma: a clinicopathological analysis of six cases Histopathology 1995 27 355 360 10.1111/j.1365-2559.1995.tb01526.x 8847066

[B15] Miettinen M Shekitka KM Sobin LH Schwannomas in the colon and rectum: a clinicopathologic and immunohistochemical study of 20 cases Am J Surg Pathol 2001 25 846 855 10.1097/00000478-200107000-00002 11420455

[B16] Daimaru Y Kido H Hashimoto H Enjoji M Benign schwannoma of the gastrointestinal tract: a clinicopathologic and immunohistochemical study Hum Pathol 1988 19 257 264 10.1016/S0046-8177(88)80518-5 3126126

[B17] Nabeya Y Watanabe Y Tohnosu N Yamazaki M Matsuda M Akutsu N Diffuse schwannoma involving the entire large bowel with huge extramural development: report of a case Surg Today 1999 29 637 641 10.1007/BF02482991 10452243

[B18] Iwamoto CA Garcia CF Razzak M Pathologic quiz case: A 23-year-old woman with a polypoid gastric mass Arch Pathol Lab Med 2003 127 e43 e44 12562296 10.5858/2003-127-e43-PQC2YO

[B19] Miettinen M Lasota J Gastrointestinal stromal tumors – definition, clinical, histological, immunohistochemical, and molecular genetic features and differential diagnosis Virchows Arch 2001 438 1 12 10.1007/s004280000338 11213830

[B20] Jacobson BC Hirsch MS Lee JH Van Dam J Shoji B Farraye FA Multiple asymptomatic plexiformschwannomas of the sigmoid colon: a case report and review Gastrointest Endosc 2001 53 801 804 10.1067/mge.2001.115317 11375596

[B21] Balaton AJ Coindre JM Cvitkovic F Tumeursstromales digestives Gastroenterol Clin Biol 2001 25 473 482 11521101

